# Residential radon and environmental burden of disease among Non-smokers

**DOI:** 10.1186/s40557-016-0092-5

**Published:** 2016-03-15

**Authors:** Juhwan Noh, Jungwoo Sohn, Jaelim Cho, Dae Ryong Kang, Sowon Joo, Changsoo Kim, Dong Chun Shin

**Affiliations:** Department of Preventive Medicine, Yonsei University College of Medicine, 50 Yonsei-ro, Seodaemun-gu, Seoul, 03722 Republic of Korea; Department of Occupational and Environmental Medicine, Gachon University Gil Hospital, Incheon, South Korea; Department of Humanities and Social Medicine, Ajou University School of Medicine, Suwon, South Korea; Institute for Environmental Research, Yonsei University College of Medicine, Seoul, South Korea

**Keywords:** Residential radon, Lung cancer, Burden of disease, Korea

## Abstract

**Background:**

Lung cancer was the second highest absolute cancer incidence globally and the first cause of cancer mortality in 2014. Indoor radon is the second leading risk factor of lung cancer after cigarette smoking among ever smokers and the first among non-smokers. Environmental burden of disease (EBD) attributable to residential radon among non-smokers is critical for identifying threats to population health and planning health policy.

**Methods:**

To identify and retrieve literatures describing environmental burden of lung cancer attributable to residential radon, we searched databases including Ovid-MEDLINE, -EMBASE from 1980 to 2016. Search terms included patient keywords using ‘lung’, ‘neoplasm’, exposure keywords using ‘residential’, ‘radon’, and outcomes keywords using ‘years of life lost’, ‘years of life lost due to disability’, ‘burden’. Searching through literatures identified 261 documents; further 9 documents were identified using manual searching. Two researchers independently assessed 271 abstracts eligible for inclusion at the abstract level. Full text reviews were conducted for selected publications after the first assessment. Ten studies were included in the final evaluation.

**Review:**

Global disability‐adjusted life years (DALYs)(95 % uncertainty interval) for lung cancer were increased by 35.9 % from 23,850,000(18,835,000-29,845,000) in 1900 to 32,405,000(24,400,000-38,334,000) in 2000. DALYs attributable to residential radon were 2,114,000(273,000-4,660,000) DALYs in 2010. Lung cancer caused 34,732,900(33,042,600 ~ 36,328,100) DALYs in 2013. DALYs attributable to residential radon were 1,979,000(1,331,000-2,768,000) DALYs for in 2013. The number of attributable lung cancer cases was 70-900 and EBD for radon was 1,000-14,000 DALYs in Netherland. The years of life lost were 0.066 years among never-smokers and 0.198 years among ever-smoker population in Canada.

**Conclusion:**

In summary, estimated global EBD attributable to residential radon was 1,979,000 DALYs for both sexes in 2013. In Netherlands, EBD for radon was 1,000–14,000 DALYs. Smoking population lost three times more years than never-smokers in Canada. There was no study estimating EBD of residential radon among never smokers in Korea and Asian country. In addition, there were a few studies reflecting the age of building, though residential radon exposure level depends on the age of building. Further EBD study reflecting Korean disability weight and the age of building is required to estimate EBD precisely.

**Electronic supplementary material:**

The online version of this article (doi:10.1186/s40557-016-0092-5) contains supplementary material, which is available to authorized users.

## Background

There were an estimated 1.8 million incident cases of tracheal, bronchus, and lung cancer (TBL cancer) and 1.6 million deaths in 2013. TBL cancer was the first cause of cancer mortality as well as the second highest absolute incidence globally in 2014 [1]. Furthermore age-standardized rates were the first in both incidence and death. In Korea, it is the third cause of cancer incidence with 15,376 cases in males and the fifth cancer with 6,751 cases in females in 2012 [2]. In Korea, the most frequent neoplasm mortality is TBL cancer in both sexes in 2014 [3].

International agency for research on cancer (IARC) classified many risk factors as the carcinogenic agents with sufficient evidence in humans including tobacco smoking, radon-222 and its decay, and second hand smoke. In the outdoor air, radon gas from the soil is diluted to low concentrations with a negligible threat to health [4]. However, indoor radon can accumulate to high levels when it confined to enclosed spaces with poor ventilation. Residential radon is the second leading risk factor of lung cancer after cigarette smoking among ever smokers and the first among non-smokers (Additional file 1: Table S1) [4]. Recently, a systematic review on the carcinogenicity of the residential radon in never smokers concluded that there seems to be an association based on few of the included studies which mostly did not focus on the association among never smokers [5].

Understanding the disease burden among major health problems and the transition pattern of the burden over time is also critical for planning global and national health policy. If a public policy addresses the major causes of disease burden, the policy could effectively contribute to the health of populations. For example, mass prevention will be possible even tiny reductions of population exposure which affect incident rate of emerging burden [6].

In addition, the principal advantage of comprehensive quantification of disease burden attributable to risk factors, particularly of modifiable factors, is critical for identifying large threats to population health and that it provides the evidence base for prevention strategies. The Global Burden of Disease (GBD) study organized risk factors into three categories; behavioral, environmental and occupational, and metabolic risks. Environmental burden of disease (EBD) is a disability‐adjusted life year (DALY) attributable to the environmental risk factors exposure. EBD is also a tool to monitor the priority of environmental risks and to make policies for the environmental health, and to evaluate the outcome of these policies afterwards.

The objective of the present study is a review of the theme of EBD of residential radon among non-smokers.

## Methods

### Search strategy

To identify and retrieve all relevant literature describing environmental burden of lung cancer attributable to residential radon, we searched databases including Ovid-MEDLINE (1948 to 5 January, 2016), Ovid-EMBASE (1980 to 5 January, 2016). Language restrictions did not apply to any of the searches. Search terms included patient keywords using ‘cancer’, ‘neoplasm’, exposure keywords using ‘residential’, ‘radon’, and outcomes keywords using ‘years of life lost’, ‘years of life lost due to disability’, ‘years lived with disability’, ‘burden’, ‘cost’. All 261 abstracts were reviewed using a combination of the search terms. Literature searches are summarized in Additional file 1: Table S1. We completed searches on January 5, 2016.

### Study selection

**Fig. 1:**
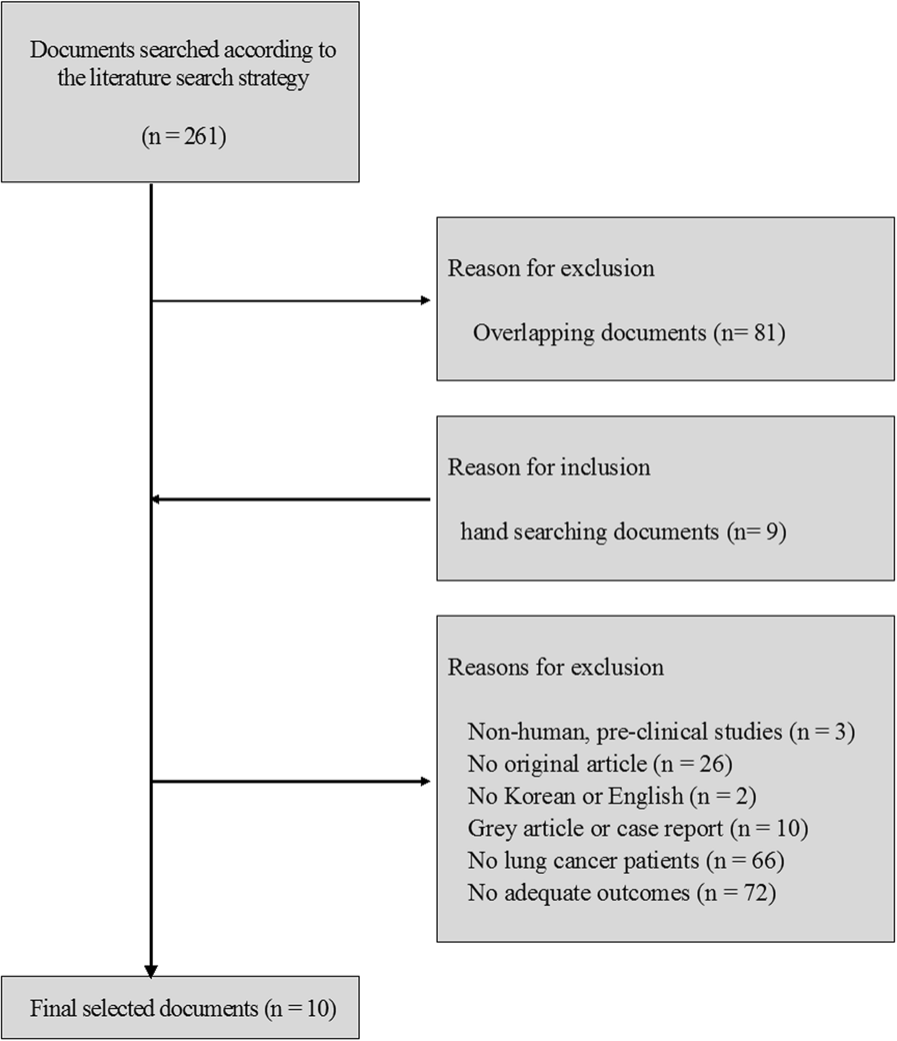
Documents selected for evaluation of the assessment according to the literature search strategy

Two researchers independently assessed publications considered to be eligible for inclusion at the title and/or abstract level. Full text reviews were conducted for the selected publications after the first assessment. Studies were included: 1) suspected lung cancer patients; 2) appropriate outcomes were reported. We excluded: 1) non-human, pre-clinical studies; 2) studies that are not written in English or Korean; 3) studies that are not original articles, gray literature, case reports. Searching through the literature identified 261 documents; 9 documents were identified using manual searching. Among these, 179 documents met our exclusion criteria. 81duplicated data from other reports were also excluded. A total of 10 studies were included in the final evaluation (Fig. 1, Additional file 1: Table S3).

## Review

Disease burden for the population could be estimated by the objective method that captures both the severity of illness and premature mortality. In 1990, GBD study proposed DALYs as a summary metric of population health [7, 8]. DALYs are a health gap between the current state of a population’s health and the goal of standard life expectancy in ideal health. No systematic reassessment of disease-specific burden worldwide has been done since the 1990 study. In the GBD 2010 results on health loss related to specific diseases and risk factors were reported: prevalence and prevalence rates for sequelae, years lived with disability (YLDs), deaths and death rates, years of life lost due to premature mortality (YLLs), and DALYs for 21 regions and worldwide in 1990, 2005 and 2010.

YLDs were calculated as the prevalence of sequelae of lung cancer by age, sex, and weighted by disability weights for lung cancer. Prevalence estimation for sequelae begins with a systematic review of all available data sources for incidence, remission, prevalence, and excess mortality. A Bayesian meta-regression method developed for the GBD 2010 (DisMod-MR) have been used to make all age-sex-country-year-specific estimates. The DisMod-MR is a generalized negative binomial model with age-standardized fixed effects and country level random effects [9]. The disability weights of GBD 1990 relied on judgments of health-care professionals; however, those of GBD 2010 derived from judgments of the general public about health severity.

YLLs were calculated from age-, sex-, country-, and time-specific mortality estimates by lung cancer, with death by standardized lost life expectancy at each age with reference to a new reference-standard life expectancy at each age. In the GBD 1990, DALYs were computed using 3 % discount rate and age weights that placed on the base case for emphasizing health outcomes in young adults. However neither YLLs nor YLDs were discounted or age-weighted in the GBD 2010. DALYs for lung cancer, as the sum of YLDs and YLLs, were calculated for sexes, 20 age groups, and 187 countries specific estimates, and aggregated estimates three points in time. Standard simulation methods were used to maintain the consistency between the sum of cause-specific mortality and independently assessed levels of all-cause mortality derived from demographic sources [9, 10]. Uncertainty around DALYs was incorporated in levels of all-cause mortality, cause-specific mortality, prevalence, and disability weights in each age-sex-country-year. Standard simulation methods by taking 1000 draws from the uncertainty distribution of RR, prevalence of residential radon exposures, TMRED, and lung cancer estimates, disability weights for each age, sex, country, year simulation analysis was used to capture uncertainty in lung cancer prevalence and death model predictions. Of the 1000 draws, the 25th and 975th value were reported as the 95 % uncertainty intervals (95 % UI).

Global DALYs due to all cause remained stable from 1990 (2,502,601,000) to 2010 (2,490,385,000). DALYs (95 % Uncertainty Interval) for lung cancer were increased by 35.9 % from 23,850,000 (18,835,000–29,845,000) DALYs in 1900 to 32,405,000 DALYs (24,400,000–38,334,000) in 2000. Crude DALYs were decreased by 23 % (4,720,500,000 to 3,614,500,000). Those for lung cancer increased by 4.5 % from 45,000,000 (35,500,000–56,300,000) to 47,000,000 (35,400,000–55,600,000) [11]. According to the order based on the mean DALYs, lung cancer increased from 24^th^ to 22^nd^ rank. There was heterogeneity in rankings of lung cancer burden among regions (Additional file 1: Table S4). DALYs for Lung cancer had high rates in the high-income regions [11].

The GBD 2010 was the first study to quantify uncertainty as the strength of the evidence on the burden of lung cancer. In addition, a systematic analysis of the proportion of disease burden caused by 67 specific risk factors including residential radon was undertaken to assess changes over time from 1990 to 2010 [12]. Disease burden of lung cancer attributable to residential radon was estimated following five steps: 1) selection of outcomes based on criteria about causal associations 2) modeling approach to estimate exposure distributions for each countries, sex, and age group; 3) estimation of relative risk per unit exposure for residential radon; 4) the choice of optimum exposure distribution, which was termed theoretical-minimum-risk exposure distribution (TMRED); 5) computation of burden attributable to residential radon by comparing the present distribution of exposure to the TMRED for each sex, and age group with 95 % UI.

Selected outcomes were trachea, bronchus, and lung cancers. The main data source for exposure was direct household measurements from surveys. The modeling approach used for estimation of current residential radon exposure distributions was mixed effect regression. The prediction of exposure distributions was done with testing many covariates, which were collected in the database at the Institute for Health Metrics and Evaluation for GBD 2010. The source of effect size per unit exposure was the publication of Darby and colleagues [13]. TMRED of residential radon was 10 Bq/m^3^. By comparing the present distribution of exposure to the TMRED for each age group, sex in 2010, the burden attributable to continuous residential radon exposure was calculated according to the following formula.

$$ \mathrm{P}\mathrm{A}\mathrm{F}=\frac{{\displaystyle {\int}_{x=0}^m}RR(x)\cdot P1(x)dx-{\displaystyle {\int}_{x=0}^m}RR(x)\cdot P2(x)dx}{{\displaystyle {\int}_{x=0}^m}RR(x)\cdot P1(x)dx} $$

RR (x) is RR at exposure level x, P1(x) is the (measured or estimated) population distribution of radon exposure, P2(x) is TMRED, which is the counterfactual distribution of exposure, and m is the maximum exposure level.

Estimated global deaths and DALYs attributable to residential radon were 98,992 (95 % UI 13,133–215,237) deaths and 2,114,000 (273,000–4,660,000) DALYs, respectively, for both sexes in 2010 (Table 2). The effect of residential radon exposure on population health differed between sexes [12]. For instance, the fraction of disease burden attributable to residential radon for lung cancer was about 2.5 times higher for men than for women in 2010 (1,514,000 DALYs for men vs 600,000 for women). Further distribution of lung cancer mortality and disease burden attributable to residential radon reveals several patterns by age group (Table 1). Globally, ranked by the attributable burden, residential radon rankings were ranged from the 27th to the 43th (Table 2) [12].

**Table 1:** Global DALYs of lung cancer and deaths attributable to residential radon by age and sex in 2010 with 95 % UI

	DALYs (in thousands)			Deaths		
	Both sexes	Male	Female	Both sexes	Male	Female
All Ages	2,114 (273–4,660)	1,514 (191–3,383)	600 (84–1,355)	98,992 (13,133–215,237)	70,014 (9,140–154,460)	28,978 (4,098–64,387)
0–6 Days	0 (0–0)	0 (0–0)	0 (0–0)	0 (0–0)	0 (0–0)	0 (0–0)
7–27 Days	0 (0–0)	0 (0–0)	0 (0–0)	0 (0–0)	0 (0–0)	0 (0–0)
28–364 Days	0 (0–0)	0 (0–0)	0 (0–0)	0 (0–0)	0 (0–0)	0 (0–0)
1–4 Years	0 (0–0)	0 (0–0)	0 (0–0)	0 (0–0)	0 (0–0)	0 (0–0)
5–9 Years	0 (0–0)	0 (0–0)	0 (0–0)	0 (0–0)	0 (0–0)	0 (0–0)
10–14 Years	0 (0–0)	0 (0–0)	0 (0–0)	0 (0–0)	0 (0–0)	0 (0–0)
15–19 Years	0 (0–0)	0 (0–0)	0 (0–0)	0 (0–0)	0 (0–0)	0 (0–0)
20–24 Years	0 (0–0)	0 (0–0)	0 (0–0)	0 (0–0)	0 (0–0)	0 (0–0)
25–29 Years	11 (1–27)	7 (1–18)	4 (0–10)	179 (19–460)	118 (12–311)	61 (6–174)
30–34 Years	20 (2–48)	12 (1–31)	7 (1–20)	361 (42–894)	227 (26–564)	134 (17–371)
35–39 Years	40 (5–100)	26 (3–62)	15 (2–37)	815 (97–2,018)	516 (58–1,250)	298 (36–756)
40–44 Years	88 (11–209)	57 (6–135)	31 (4–74)	1,979 (252–4,700)	1,284 (142–3,042)	696 (96–1,665)
45–49 Years	154 (20–352)	105 (13–243)	48 (7–112)	3,884 (506–8,889)	2,662 (324–6,136)	1,222 (168–2,836)
50–54 Years	247 (32–544)	177 (22–393)	70 (9–157)	7,079 (921–15,575)	5,081 (638–11,274)	1,998 (267–4,503)
55–59 Years	343 (44–785)	253 (31–580)	90 (14–205)	11,324 (1,463–25,961)	8,358 (1,026–19,168)	2,965 (446–6,775)
60–64 Years	348 (44–774)	258 (33–570)	90 (12–201)	13,548 (1,724–30,220)	10,053 (1,288–22,176)	3,494 (477–7,851)
65–69 Years	306 (39–667)	225 (29–503)	82 (12–181)	14,401 (1,845–31,358)	10,552 (1,339–23,633)	3,849 (553–8,534)
70–74 Years	271 (36–586)	196 (26–426)	74 (11–165)	15,897 (2,099–34,473)	11,536 (1,533–25,026)	4,362 (630–9,719)
75–79 Years	181 (25–398)	128 (17–282)	53 (8–123)	13,896 (1,932–30,443)	9,792 (1,312–21,581)	4,104 (597–9,477)
80+ Years	106 (15–223)	70 (10–148)	37 (5–81)	15,630 (2,235–32,630)	9,834 (1,413–20,831)	5,796 (841–12,628)

*DALYs* disability-adjusted life-years, *UI* uncertainty intervals

**Table 2:** Global deaths, DALYs for both sexes attributable to residential radon in 1990, 2010, and 2013 with 95 % UI

	All risk factors	Environmental risks	Residential radon
1990 deaths (in thousands)	25,085 (24,385 to 25,821)	8,492 (8,036 to 8,953)	63 (41 to 86)
2010 deaths (in thousands)	N/A	N/A	99 (13–215)
2013 deaths (in thousands)	30,839 (29,719 to 31,949)	8,181 (7,651 to 8,726)	92 (61 to 128)
Median percent change deaths	23.0 % (19.0 to 27.3)	−3.7 % (−9.6 to 2.4)	46.3 % (13.1 to 87.9)
Median percent change of age standardized deaths PAF	0.6 % (−1.0 to 2.0)	−15.5 % (−19.8 to 11.1)	13.8 % (−11.7 to 44.3)
1990 DALYs (in thousands)	1,035,987 (980,813 to 1,092,478)	400,345 (374,489 to 424,432)	1,503 (984 to 2,086)
2010 DALYs (in thousands)	2,490,385 (95 % UI NA)	N/A	2,114 (2,73–4660)
2013 DALYs (in thousands)	996,554 (927,157 to 1,072,340)	289,517 (265,778 to 312,094)	1,979 (1,331 to 2,768)
Median percent change DALYs	−3.8 % (−7.7 to −0.1)	−27.7 % (−32.1 to −23.2)	31.7 % (2.4 to 67.6)
Median percent change of age standardized DALYs PAF	−3.8 % (−6.0 to −1.8)	−22.6 % (−26.1 to −19.1)	7.1 % (−17.0 to − 36.9)

*DALYs* disability-adjusted life-years, *PAF* population attributable fraction, *UI* uncertainty intervals

The estimates in 1990 used the result of GBD 2013

Percent change is the change of estimates between 1990 and 2013

The strengths of the study by Lim SS et al [12]. included the incorporation of more data for residential radon exposure, sophisticated methods to handle missing data, comparability of methods, use of TMRED as the counterfactual distribution with which observed exposures are compared, and global patterns with regional variation in risks to lung cancer. Like other disease burden studies, there were also some limitations. First, in the estimates of effect sizes, residual confounding cannot be definitively adjusted. Second, the external validity of the effect size to diverse population still remains questionable due to uncertainty. Third, the effect size could change over time that effect size integrated using evidences across different periods could not reflect temporal change in residential radon. Fourth, the assumption that all other factors were independent, in the quantification of attributable burden of residential radon, could be incorrect. Fifth, there were not any DALYs attributable to residential radon for subpopulation by the smoking status.

In 2015, GBD study 2013 reassessed the risk factors with new data for exposure, relative risks, and evidence on the counterfactual radon exposure distribution [14, 15]. In general, the analysis used the comparative risk assessment methods of the GBD 2010. The estimates of attributable deaths, YLL, YLD, and DALYs of lung cancer have been estimated for 79 risks or clusters of risks, including residential radon. Conceptually, the amount of lung cancer disease burden observed in a given year was calculated and that attributable to past exposure to residential radon was evaluated.

The attributable burden for radon in the specific sex *s*, age group *a*, country *c* and year *t* (AB_*Rn_asct*_) was estimated as the sum, of DALYs for cause lung cancer (DALY_*LC_asct*_) multiplied by PAF for lung cancer due to indoor radon (PAF_*Rn_LC_asct*_) following the equation.

$$ A{B}_{Rn\_ asct}={\displaystyle \sum^W}{\mathrm{DALY}}_{LC\_ asct}\cdot {\mathrm{PAF}}_{Rn\_LC\_ asct}={\displaystyle \sum^W}\left({\mathrm{YLL}}_{LC\_ asct}+{\mathrm{YLD}}_{LC\_ asct}\right)\cdot {\mathrm{PAF}}_{Rn\_LC\_ asct} $$

The PAF_*Rn_LC_asct*_ was defined in each group was defined as

$$ {\mathrm{P}\mathrm{AF}}_{Rn\_LC\_ asct}=\frac{{\displaystyle {\int}_{x=l}^u}R{R}_{Rn\_LC\_asc}(x)\cdot {\mathrm{P}}_{Rn\_ asct}(x)dx-R{R}_{Rn\_LC\_asc}\left( TMRE{L}_{Rn\_as}\right)}{{\displaystyle {\int}_{x=l}^u}R{R}_{Rn\_LC\_asc}(x)\cdot {\mathrm{P}}_{Rn\_ asct}(x)dx} $$

RR_*Rn_LC_asc*_ (x) is a function of exposure level x for risk factor residential radon, lung cancer, age-group *a*, sex *s*, and country *c*. P_*Rn_asct*_ (x) is the radon exposure distribution function for in age-group *a*, sex *s*, country *c*, and year *t. l* is the lowest level and *u* is the highest level of exposure observed. TMREL_Rn_as_ is the theoretical minimum radon exposure level, which is the counterfactual level of risk exposure that is minimum overall risk theoretically possible to achieve, for age group *a*, and sex *s* and the single level of radon exposure that minimize risk from all cause of DALYs.

The exposure of residential radon was defined as the average daily exposure to indoor air radon levels measured in Becquerel (radon disintegrations per s) per cubic meter (Bq/m^3^) and the TMREL was 10 Bq/m^3^, the outdoor concentration of radon. New country-level average data for radon exposure were added after the GBD 2010 and the modeling process was also updated. The methodology to synthesize radon exposure data has been moderately changed from a mixed effects regression model to spatiotemporal Gaussian process regression (ST-GPR). Only for risk factors with sufficient data in standard age groups or at the household level, ST-GPR has been used to estimate a flexible time trend. The RR and TMREL were not updated for the GBD 2013.

At the global level, the correlation of the DALYs attributable to the same risks for the year 2010 between GBD 2010 and GBD 2013 was 0.97. Estimated global deaths and DALYs attributable to residential radon were 92,000 (95 % UI 61,000–128,000) deaths and 1,979,000 (1,331,000–2,768,000) DALYs, respectively, for both sexes in 2013.

Similar study for the burden in the United States was published in 2013. US Burden of Disease Collaborators estimated burden of diseases, injuries, and risk factors, which included environmental risk factors [16]. Following the conceptual framework for risk factors developed for the GBD [17], authors estimated the deaths or DALYs related to the environmental causes including residential radon. The process of computation was consists of 3 key steps. The first step was inclusion of the risk-outcome pairs. Residential radon was “convincing” or “probable” for only lung cancer according to the grading system defined by the World Cancer Research Fund [18]. Based on systematic reviews and meta-analyses of relative risks for diseases due to specific risks and exposure researches, deaths and DALYs were related to risk factors. Relative risk of lung cancer due to residential radon exposure for both sexes aged 0-80 + years was 1.002 (1.00 - 1.003) per 1 Bq/m^3^. In the second step, the age-, sex- specific distribution of residential radon exposure was estimated from other published data sources [12]. The third step was the estimation of deaths or DALYs associated with residential radon by comparing the current distribution of exposure with TMRED of exposure selected for each risk factor. The age-standardized DALY and all age DALY of lung cancer due to residential radon was 183,200 (18,800 – 514,200) and 4,200,000 (430,000 – 11,720,000), respectively in 2010.

In 2013, the study of burden of disease related to indoor air in Netherlands [19] reported that the second largest most important indoor air pollutants were radon and thoron from soils and building materials following environmental tobacco smoke. Lung cancer as health effect was selected because there was ‘sufficient evidence of an association’ with the indoor radon according to WHO and meta-analysis [20, 21]. All Dutch inhabitants were considered as the population exposed to indoor radon. The level of radon exposure was based on measurements in newly built homes, which estimated that the average indoor radiation dose due to radon is 0.39 mSv per year in the Netherlands. The exposure − effect relationship, in BEIR-VI [22], stratified by smoking status was used to quantify the effects size of radon exposure. PAF was calculated following equations.

$$ \mathrm{A}\mathrm{F}=\frac{\left(RR-1\right)}{RR} $$

$$ \mathrm{P}\mathrm{A}\mathrm{F}=\mathrm{A}\mathrm{F}\cdot \mathrm{F} $$

$$ \mathrm{AB}=\mathrm{P}\mathrm{A}\mathrm{F}\cdot \mathrm{P} $$

$$ \mathrm{D}\mathrm{ALY}=\mathrm{AB}\cdot \mathrm{D}\cdot \mathrm{S} $$

RR is a relative risk of 0.39 mSv radon exposure per year. F is the fraction of the total population that is exposed. AB is an attributive burden, or excess cases. P is a baseline prevalence or incidence or mortality rates. D is an average duration of the lung cancer until remission in years or YLL for the lung cancer mortality. S is the severity weight of the lung cancer between 0 (healthy) and 1 (death) and it have been determined by the Dutch Disability Weights Group [23]. The lowest RR of the 95 % confidence interval was multiplied by the lowest radon exposure estimate and severity weight to calculate the lowest estimate of the lung cancer burden, and the highest estimate was obtained in a similar manner. Exposure level was 0.45–0.74 mSv/year, RRs were derived from the BEIR VI report, severity was 0.43–0.54, and duration was 1.61 years. The number of attributable lung cancer cases was 70–900. EBD for radon was 1,000–14,000 DALYs. This was higher compared to that of previous study (EnVIE), which was 1,300 DALYs [24]. It was because the 2013 Netherland study included not just soil but building materials as radon source, whereas EnVIE considered soil as the sole radon source. And EnVIE study used PAF estimated directly by the judgment of experts and disease burden from burden of disease in the WHO database, which were calculated with the standardized country-specific data to optimize comparability across countries.

In Ontario, Canada in 2013, the study of burden of lung cancer illness due to radon also calculated YLL and population attributable risk percent, excess life-time risk ratio, the number of lung cancer deaths due to radon [25]. This study provided local level research and evidence for 36 health units, varying in geographical and population size. The method documented by Brand et al. [26], based on the exposure-age-concentration model from the BEIR-VI report, was implemented. The excess risk ratio (ERR) of lung cancer mortality for age groups with 5 year intervals was calculated with health unit-specific radon exposure information and the percentage of the population living in high-rise buildings. The uncertainty in ERR was estimated with a Monte Carlo simulation in order to capture exposure uncertainty and inter-individual variability of the ERRs. In each health unit, YLL, ELRR, and PAR % were calculated following the equations.

$$ \mathrm{Y}\mathrm{L}\mathrm{L}=\mathrm{L}\mathrm{E}-{\mathrm{LE}}_E $$

$$ \mathrm{ELRR}=\frac{{\mathrm{LR}}_E}{LR-1} $$

$$ \mathrm{P}\mathrm{A}\mathrm{R}\ \%=\frac{\left({\mathrm{LR}}_E-\mathrm{L}\mathrm{R}\right)}{{\mathrm{LR}}_E} \times 100 $$

LR is lifetime risk and LE is life expectancy estimates of lung cancer for each health unit. Estimates were estimated separately for ever-smokers, never-smokers, and combined (ever- and never-smokers, assuming that 92.5 % of lung cancers are in ever-smokers [27]). Data were from the age-health unit-specific lung cancer, all-cause mortality data, age-specific proportion of ever-smokers in each health unit, and the age-specific RRs of lung cancer and all cause mortality due to smoking. LR_E_ and LE_E_ are estimates of lung cancer in exposed individuals, which were calculated by the life-table multiplied by the simulated ERRs. A population-weighted average of the health unit estimates were used to calculate PAR %, ELRR, and YLL estimates for the Ontario. The number of lung cancer deaths due to radon, in both never- and ever-smokers combined, were calculated as the mean combined PAR % multiplied by the number of lung cancer deaths in that health unit in 2007. In terms of YLL for lung cancer patients, arithmetic mean for the overall Ontario population was one-sixth of a life year lost (0.164 years). Smoking population lost three times more years than never-smokers. The arithmetic mean PAR % estimates was 13.6 %, which means lung cancer deaths within the Ontario are attributable to radon (Table 3). Never-smokers had higher PAR % values (21.9 %) than ever-smokers (12.3 %). At current levels in Ontario, there was 16 % greater risk of lung cancer compared to background levels (Table 3). In the never-smoking population, the ELRR could be 1.304 (97.5 % quintile), which was also higher than ever-smokers. In addition, 36 health units across Ontario had a considerably heterogeneous PAR % estimates.

**Table 3:** Estimated arithmetic mean PAR %, deaths, ELRR, and YLL attributable to residential radon for Ontario by smoking status

Geographical region	Smoking status	PAR %	Deaths (95 % CI)	ELRR	YLL
Ontario	Combined^a^	13.6	847 (686, 1,039)	0.161	0.164
	Never	21.9	102 (85, 124)	0.291	0.066
	Ever	12.3	708 (570, 869)	0.143	0.198
HU1 (Highest PAR %)	Combined^a^	25.3	21 (18.2, 24.9)		
	Never	40.9	3 (2.2, 2.9)		
	Ever	22.9	18 (15.0, 20.7)		
HU2 (Lowest PAR %)	Combined^a^	9.1	24 (18.8, 31.3)		
	Never	15.8	3 (2.4, 3.9)		
	Ever	8.2	20 (15.4, 25.9)		

Data are shown for Ontario and two selected health units with the highest (HU1) and lowest (HU2) PAR % values among all 36 health units. The number of deaths are rounded to the nearest whole number

*PAR %* population attributable risk percent, *ELRR* excess life-time risk ratio, *YLL* years of life lost, *CI* confidence interval

^a^Combined (Both never and ever)

The Ontario study was the first study estimating the lung cancer burden attributable to radon by health unit within a Canada. Likewise other disease burden study, limitations of the study include the representativeness of the exposure data, which was from fewer than 100 samples and radon levels vary widely for some health units. The lung cancer burden estimates could be influenced by the distribution of measured radon levels.

## Conclusion

In summary, estimated global EBD attributable to residential radon was 1,503,000 (984,000 to 2,086,000) DALYs for both sexes in GBD 1990. Estimates were 2,114,000 (273,000–4,660,000) and 1,979,000 (1,331,000–2,768,000) DALYs for both sexes in GBD 2010 and 2013, respectively. Stratified by sex, EBD estimates attributable to residential radon were 1,514,000 (191,000–3,383,000) among male and 600,000 (84,000–1,355,000) among female. In United States, the age-standardized DALY and all age DALY of lung cancer due to residential radon was 183,200 (18,800 – 514,200) and 4,200,000 (430,000 – 11,720,000), respectively in 2010. In Netherlands, EBD for radon was 1000–14,000 DALYs. Smoking population lost three times more years than never-smokers (0.066 vs 0.198) in Canada, 2013. Although it has been reported that the smoking status modifies the risk of lung cancer associated with radon, EBD among never smokers has not been explored yet. Furthermore there was no study estimating EBD of residential radon among never smokers in Korea. In addition, there were a few studies reflecting the age of building, though residential radon exposure level depends on the age of building. In conclusion, further EBD study reflecting Korean disability weight and the age of building is required to estimate EBD precisely.

## Additional file

Additional file 1: Table S1. Lifetime lung cancer incidence and comparison with other cause of death by the smoking status. Table S2. Ovid-MEDLINE, Ovid-EMBASE search strategy in 5th of Jan, 2016. Table S3. Documents selected for the systematic review. Table S4. Residential radon ranked by region and attributable burden of disease, 2010. (DOCX 29 kb)
