# Supplementary material for: Residential radon and environmental burden of disease among Non-smokers
Source: Ann Occup Environ Med. 2016 Mar 15;28:12. doi: 10.1186/s40557-016-0092-5 (PMC4793519; doi:10.1186/s40557-016-0092-5)
Supplement: Additional file 1: — Table S1. Lifetime lung cancer incidence and comparison with other cause of death by the smoking status. Table S2. Ovid-MEDLINE, Ovid-EMBASE search strategy in 5th of Jan, 2016. Table S3. Documents selected for the systematic review. Table S4. Residential radon ranked by region and attributable burden of disease, 2010. (DOCX 29 kb) [file 40557_2016_92_MOESM1_ESM.docx]

| Supplementary table 1. Lifetime lung cancer incidence and comparison with other cause of death by the smoking status | | | | |
| --- | --- | --- | --- | --- |
| Smoking status | Radon Level (pCi/L) | Lifetime lung cancer incidence * (per 1000) | The risk comparison with other risks ** | Possible intervention † |
| Smokers | 20 | 260 | 250 times the risk of drowning | Stop smoking and fix your home |
|  | 10 | 150 | 200 times the risk of dying in a home fire | Stop smoking and fix your home |
|  | 8 | 120 | 30 times the risk of dying in a fall | Stop smoking and fix your home |
|  | 4 | 62 | 5 times the risk of dying in a car crash | Stop smoking and fix your home |
|  | 2 | 32 | 6 times the risk of dying from poison | Stop smoking and consider fixing between 2 and 4 pCi/L |
|  | 1.3 | 20 | (Average indoor radon level) | (Reducing radon levels below 2 pCi/L is difficult.) |
|  | 0.4 | 3 | (Average outdoor radon level) |  |
|  |  | |  |  |
| Non-smokers | 20 | 36 | 35 times the risk of drowning | Fix your home |
|  | 10 | 18 | 20 times the risk of dying in a home fire | Fix your home |
|  | 8 | 15 | 4 times the risk of dying in a fall | Fix your home |
|  | 4 | 7 | The risk of dying in a car crash | Fix your home |
|  | 2 | 4 | The risk of dying from poison | Consider fixing between 2 and 4 pCi/L |
|  | 1.3 | 2 | (Average indoor radon level) | (Reducing radon levels below 2 pCi/L is difficult.) |
|  | 0.4 |  | (Average outdoor radon level) |  |
| * Lifetime risk of lung cancer deaths from US Environmental Protection Agency Assessment of Risks from Radon in Homes (EPA 402-R-03-003). | | | | |
| ** Comparison data calculated using the Centers for Disease Control and Prevention's 1999-2001 National Center for Injury Prevention and Control Reports. | | | | |
| † Possible intervention from US Environmental Protection Agency webpage (http://www.epa.gov/radon/health-risk-radon) | | | | |

Supplement table 2. Ovid-MEDLINE, Ovid-EMBASE search strategy in 5^th^ of Jan, 2016

|  | No. | Search term | Ovid-MEDLINE  Searched no. | Ovid-EMBASE  Searched no. |
| --- | --- | --- | --- | --- |
|  | 1 | cancer.mp OR exp neoplasm/ | 3.093.703 | 3,838,533 |
|  | 2 | lung.mp OR exp lung/ | 693.089 | 1,201,023 |
|  | 3 | 1 AND 2 | 262,555 | 417,674 |
|  | 4 | YLL.mp | 215 | 296 |
|  | 5 | years of life lost.mp | 941 | 1,106 |
|  | 6 | 4 OR 5 | 970 | 1,171 |
|  | 7 | YLD.mp | 166 | 223 |
|  | 8 | years of life lost due to disability.mp | 16 | 23 |
|  | 9 | years lived with disability.mp | 232 | 275 |
|  | 10 | 7-9 / OR | 313 | 404 |
|  | 11 | burden.mp | 131,495 | 168,023 |
|  | 12 | cost.mp | 373,329 | 620,284 |
|  | 13 | 11 OR 12 | 486,485 | 762,559 |
|  | 14 | 6 OR 10 OR 13 | 487,003 | 763,170 |
|  | 15 | residential.mp | 26,998 | 38,630 |
|  | 16 | radon.mp | 6,727 | 9,365 |
|  | 17 | 15 OR 16 | 33,354 | 47,518 |
| Total | 18 | 3 AND 14 AND 17 | 100 | 161 |

Supplementary table 3. Documents selected for the systematic review

| Research type | Author  (Publication yr) | Outcomes |
| --- | --- | --- |
|  | GBD Study 2013 collaborators (2015) | ▪ Tracheal, bronchus, and lung cancer  Prevalent cases in 2013 (per 1,000): 3,227.4 (3,039.7~3,426.8)  Percentage change in prevalence from 1990 to 2013: 72.2 (61.9~82.1)  Percentage change in age-standardized prevalence from 1990 to 2013: 0.8 (-5.4~6.5)  YLDs in 2013 (per 1,000): 467.4 (338.5~593.2)  Percentage change in YLDs from 1990 to 2013: 64.6 (54.9~72.9)  Percentage change in age-standardized YLDs from 1990 to 2013: -3.7 (-9.3~1.2) |
|  | GBD 2013 DALYs and HALE collaborators (2015) | ▪ Tracheal, bronchus, and lung cancer  All age DALYs (thousands) 2005yr: 30,791.6 (29,492.6~31,587.1)  All age DALYs (thousands) 2013yr: 34,732.9 (33,042.6~36,328.1)  All age DALYs (thousands) percentage change: 12.9 (6.6~19.1)  Age-standardized DALYs (per 100,000) 2005yr: 586.7 (562.2~601.6)  Age-standardized DALYs (per 100,000) 2013yr: 542.8 (516.4~567.1)  Age-standardized DALYs (per 100,000) percentage change: -7.4 (-12.4~-2.5) |
|  | GBD 2013 morality and causes of death collaborators | ▪ Tracheal, bronchus, and lung cancer  All ages deaths (thousands) 1990yr: 1,050.0 (1,010.6~1,078.2)  All ages deaths (thousands) 2013yr: 1,639.6 (1,565.6~1,706.0)  All ages deaths (thousands) median % change: 56.5 (47.83~62.84)  Age-standardized death rate (per 100,000) 1990yr: 29.6 (28.5~30.4)  Age-standardized death rate (per 100,000) 2013yr: 27.0 (25.7~28.1)  Age-standardized death rate (per 100,000) median % change: -8.7 (-13.62~-5.16) |
|  | GBD 2013 risk factors collaborators (2015) | ▪ Residential radon  1990 deaths (in thousands): 63 (41~86)  2013 deaths (in thousands): 92 (61~128)  median percent change deaths: 26.3% (13.1~87.9)  Median percent change of age-standardized deaths PAF: -1.9% (-11.7~44.3)  1990 DALYs (in thousands): 1,503 (984~2,086)  2013 DALYs (in thousands): 1,979 (1,331~2,768)  Median percent change DALYs: 31.7% (2.4~67.6)  Median percent change of age-standardized DALYs PAF: 7.1% (-17~-36.9) |
|  | GBD collaborators (2015) | ▪ Tracheal, bronchus and lung  Incident cases, global (thousands) total: 1798  Incident cases, global (thousands) male: 1,263  Incident cases, global (thousands) female: 535  Deaths, global (thousands) total: 1,640  Deaths, global (thousands) male: 1,155  Deaths, global (thousands) female: 485  DALYs: 34.7 million  DALYs, men: 24.9 million  DALYs, women: 9.8 million |
|  | Schram-Bijkerk *et al* (2013) | ▪ Number of attributable cases and DALYs attributable to residential radon  Lung cancer, incidence 70~900  Lung cancer, deaths: 70~900  DALYs attributable to residential radon: 1,000–14,000 |
|  | US burden of disease collaborators (2013) | ▪ Lung cancer  YLL rank 1990: 2 (2~3)  YLL rank 2010: 2 (2~2)  Death No. (in thousands) 1990yr: 143.5 (116.8~178.5)  Death No. (in thousands) 2010yr: 163.3 (128.1~200.8)  Death median change%: 14.4 (-1.1~26.0)  Death age-standardized death rate: -22.5 (-31.8~-14.7)  YLLs No. (in thousands) 1990yr: 2,871.9 (2,325.8~3,523.2)  YLLs No. (in thousands) 2010yr: 2,987.7 (2,418.1~3,731.2)  YLLs median change%: 3.6 (-6.6~17.4)  YLLs age-standardized death YLL:-30 (-36.1~-18.9) |
|  | Peterson *et al* (2013) | ▪ Lung cancer deaths attributable to radon among never smokers (95% CI)  Ontario: 102 (85~124)  HU1: 3 (2.2~2.9)  HU2: 3 (2.4~3.9) |
|  | Murray *et al* (2012) | ▪ Tracheal, bronchus and lung  All ages DALYs (thousands) 1990yr: 23,850 (18,835~29,845)  All ages DALYs (thousands) 2010yr: 32,405 (24,400~38,334)  All ages DALYs (thousands) percentage change: 35.9  DALYs (per 100,000) 1990yr: 450 (355~563)  DALYs (per 100,000) 2010yr: 470 (354~556)  DALYs (per 100,000) percentage change: 4.5 |
|  | Lim *et al* (2012) | ▪ Deaths attributable to residential radon (GBD 2010)  Men: 70,014 (9,140~154,460)  Women: 28,978 (4,098~64,387)  Both sexes: 98,990 (13,133~215,237) |

1. Global, regional, and national incidence, prevalence, and years lived with disability for 301 acute and chronic diseases and injuries in 188 countries, 1990–2013: a systematic analysis for the Global Burden of Disease Study 2013

2. Global, regional, and national disability-adjusted life years (DALYs) for 306 diseases and injuries and healthy life expectancy (HALE) for 188 countries, 1990–2013: quantifying the epidemiological transition

3. Global, regional, and national age-sex specific all-cause and cause-specific mortality for 240 causes of death, 1990-2013: a systematic analysis for the Global Burden of Disease Study 2013

4. Global, regional, and national comparative risk assessment of 79 behavioral, environmental and occupational, and metabolic risks or clusters of risks in 188 countries, 1990-2013: a systematic analysis for the Global Burden of Disease Study 2013

5. The Global Burden of Cancer 2013

6. The burden of disease related to indoor air in the Netherlands: Do different methods lead to different results?

7. The State of US health, 1990-2010: Burden of diseases, injuries, and risk factors

8. Lung cancer risk from radon in Ontario, Canada: how many lung cancers can we prevent?

9. Disability-adjusted life years (DALYs) for 291 diseases and injuries in 21 regions, 1990-2010: a systematic analysis for the Global Burden of Disease Study 2010

10. A comparative risk assessment of burden of disease and injury attributable to 67 risk factors and risk factor clusters in 21 regions, 1990-2010: a systematic analysis for the Global Burden of Disease Study 2010

| Supplementary table 4. Residential radon ranked by region and attributable burden of disease, 2010 | |
| --- | --- |
| Regions | Ranking |
| Global | 32 |
| High-income Asia Pacific | 27 |
| Western Europe | 35 |
| Australasia | 27 |
| High-income North America | 28 |
| Central Europe | 36 |
| Southern Latin America | 33 |
| Eastern Europe | 32 |
| East Asia | 36 |
| Tropical Latin America | 41 |
| Central Latin America | 41 |
| Southeast Asia | 38 |
| Central Asia | 42 |
| Andean Latin America | 41 |
| North Africa and Middle East Caribbean | 42 |
| South Asia | 41 |
| Oceania | 42 |
| Southern sub-Saharan Africa | 42 |
| Eastern sub-Saharan Africa | 43 |
| Central sub-Saharan Africa | 43 |
| Western sub-Saharan Africa | 43 |
| Regions are ordered by mean life expectancy | |
| In 2013, residential radon was not ranked in the ten leading in any region | |
